# Impact of a 7-day short peptide diet on gut microbiota and metabolomics in septic mice

**DOI:** 10.3389/fnut.2025.1522429

**Published:** 2025-02-25

**Authors:** Dan Zuo, Binyu Zuo, Liuyang Wang, Dabi Hu, Yang Yang, Yong Chen, Biao Huang

**Affiliations:** ^1^Clinical Nutrition Department, The Affiliated Dazu’s Hospital of Chongqing Medical University, Chongqing, China; ^2^School of Stomatology, Xinjiang Medical University, Xinjiang, China; ^3^Department of Critical Care Medicine, The First Affiliated Hospital of Chongqing Medical University, Chongqing, China; ^4^Department of Critical Care Medicine, The Affiliated Dazu’s Hospital of Chongqing Medical University, Chongqing, China; ^5^The Chongqing Key Laboratory of Translational Medicine in Major Metabolic Diseases, Chongqing, China

**Keywords:** short peptides, sepsis, intestinal flora, intestinal metabolites, metabolomics

## Abstract

**Objective:**

Our study aim is to explore the mechanisms of short peptide passages on intestinal dysfunction in septic mice utilizing a metabolomics approach, which provides a new scientific basis for the clinical study of sepsis.

**Methods:**

Mices were allocated at random into four groups: control (Con), cecal ligation and puncture followed by one, three or 7 day short-peptide-based enteral nutrition group (CLP + SPEN1), (CLP + SPEN3), and (CLP + SPEN7) groups. A liquid chromatography-mass spectrometry-based metabolomics method was used to analyze changes in serum metabolites in septic mice.

**Results:**

Short peptides showed effectiveness in reducing symptoms, mucosal inflammation, and intestinal function damage scores in septic mice. The 16sRNA analysis showcased significant variances in the distribution of bacterial communities between the CLP + SPEN1, CLP + SPEN3, and CLP + SPEN7 groups. At the phylum level, statistically significant variances in the relative abundance of Proteobacteria, Firmicutes, and Bacteroidetes were recognized. The metabolomics analysis results showed significant separation of metabolites between the CLP + SPEN1 and CLP + SPEN3 groups, as well as significant differences in metabolite profiles between the CLP + SPEN3 and CLP + SPEN7 groups. Utilizing a differential Venn diagram, four metabolites were commonly different; 10-heptadecanoic and dodecanoic acids had statistical significance. The abundance of both dodecanoic and lactic acid bacteria was negatively associated at the genus level.

**Conclusion:**

Short peptides were found to promote the growth of beneficial bacteria, Lactobacillus and uncultured_bacterium_f_Muribaculaceae, while reducing intestinal metabolites such as Dodecanoic acid and 10-Heptadecenoic acid. Moreover the Lactobacillus may play a significant therapeutic role in the treatment of sepsis. However, due to the limited number of experimental samples, the exact mechanism of action of short peptides awaits further confirmation.

## 1 Introduction

Sepsis represents a life-threatening disease characterized by endothelial barrier dysfunction and impaired normal microcirculatory function, leading to a state of inadequate perfusion and tissue edema (1); furthermore, it is identified by acute organ failure that impacts more than 30 million people globally (2). Sepsis and septic shock are major international public health problems. The World Health Organization (WHO) stated that almost 189 deaths cases occur per 100,000 in-hospital sepsis patients (3). The incidence of sepsis has risen since the first consensus definition was established in 1991, and there is a great need to improve the understanding of sepsis and provide better treatment (4). In 2017, WHO member states announced enhancing sepsis prevention, recognition, and treatment as a global health priority (5).

The gastrointestinal tract has been recognized for a long time as an integral part of the pathophysiology of sepsis, and gut microbiota (GM) aberrations may be the key factor (6). The maintenance of intestinal barrier integrity supports homeostasis, and in principle, any infection or severe trauma may lead to significant alterations in intestinal barrier homeostasis. Sepsis commences with infection and finally results in cytokine storms, capillary endothelial damage, capillary leakage, microthrombosis, and reduced tissue perfusion, resulting in organ dysfunction. Inadequate perfusion of visceral regions is considered to be one of the main causes of mucosal intestinal barrier disruption during sepsis (7). Sepsis significantly alters the composition and distribution of the intestinal flora, which creates the conditions for the onset and progression of sepsis (8). Sepsis has been reported to cause imbalances in the intestinal microecosystem, including reduced microbial diversity and abundance (9). Therefore, improving intestinal barrier damage is considered an important measure in the treatment of sepsis. Early enteral nutrition (EN) enhances gastrointestinal blood flow, protects intestinal mucosal structures, stimulates enzymatic processes, and improves systemic immune responses (10).

Peptides are compounds formed by *α*-amino acids linked together in a peptide chain. They are also intermediate products of protein hydrolysis. Bioactive peptides from various food sources have been proven to have health promoting effects, and food derived peptides have the potential to alter intestinal barrier function (IBF) and promote disease treatment (11). The experimental results from Professor Hyosuk Son’s team showed that all peptides inhibited the expression of LPS-induced NO and cytokines in RAW 264.7 cells by blocking the interaction between LPS and macrophages. Antimicrobial peptides (AMPs) possess anti-bacterial and anti-inflammatory activity, neutralizing toxins such as lipopolysaccharides (LPS, endotoxin) (12). Short peptides are receiving increasing attention in the fields of biology, chemistry, and medicine due to their unique properties. They are considered to be innovative therapeutic agents capable of improving efficacy while minimizing potential side effects. However, the impacts of short peptide enteral nutrition preparations on intestinal flora and intestinal metabolites are still unknown. Therefore, the cecum ligation and puncture sepsis mouse model was utilized to assess the therapeutic impacts of short peptides on septic mice, and to investigate. The impacts of short peptide enteral nutrition preparations on intestinal flora and metabolites.

## 2 Materials and methods

### 2.1 Main reagents

Short peptide enteral nutrition (Nutricia, China), 4% paraformaldehyde (Biosharp, USA), PBS (Lianke Biotechnology, China), Anti IL-10 monoclonal antibody (Lianke Biotech, China), Anti TNF-a monoclonal antibody (PeproTech, USA), Anti IL-6 monoclonal antibody (PeproTech, USA), RNAiso Pls (Takara, Japan), NA Reverse Transcription Kit (Takara, Japan), PCR Primers (Takara, Japan),SYBR Premix Ex Taq (Takara, Japan), DEPC water (Takara, Japan), 3-phosphoglyceraldehyde dehydrogenase (GAPDH) (Takara, Japan), PrimeScript ™ RT reagent kit with gDNA Eraser (Takara, Japan), TB Green^®^ Premix Ex Taq^™^ II (Takara, Japan), SDS-PAGE gel rapid preparation kit (Biyuntian, China), BCA protein concentration assay kit (Biyun Tian, China), SDS-PAGE protein loading buffer (Biyuntian, China), RIPA cracking solution (Biyun Tian, China), MUC2 antibody (Abcam, UK), ZO-1 antibody (Abcam, UK), Occludin-1 (Abcam, UK), Chloroform (Chuandong Chemical, China), Isopropanol (Chuandong Chemical, China), Anhydrous ethanol (Chuandong Chemical, China), Formaldehyde (Chuandong Chemical, China), Sodium formate (Jiangshan Biotechnology Co., Ltd., China), Ammonium formate (Jiangshan Biotechnology Co., Ltd., China), Anticoagulant blood collection tube (Biyun Tian, China), Glutaraldehyde (Jiangshan Biotechnology Co., Ltd., China), Pyridine (Jiangshan Biotechnology Co., Ltd., China),Acetonitrile (Jiangshan Biotechnology Co., Ltd., China).

### 2.2 Main instruments and equipment

Pipette gun (Eppendorf, Germany), Low temperature high-speed centrifuge (Thermo Fisher, USA), Room temperature centrifuge (ordinary instrument, China), Disposable 1 mL sterile syringe (BD, USA), Disposable 3 mL Babbitt tube (Nest, China), Disposable 15 mL centrifuge tube (Nest, China), Disposable 50 mL centrifuge tube (Nest, China), Ultra low temperature refrigerator (Thermo Fisher, USA), Ordinary refrigerator (Haier, China), Electron microscope (Leica, Germany), Multi functional enzyme-linked immunosorbent assay (Bio Rad, USA), RM2135 Slicer (Leica, Germany), Microwave Oven (Galanz, China), Upright fluorescence microscope (Nikon, Japan), Fluorescent quantitative PCR instrument (Bio Rad, USA), Decolorization shaker TS-100 (Qilin Bell, China), Electrophoretic apparatus (Bio Rad, USA), Gel imager (Bio Rad, USA).

### 2.3 Preparation of main reagents

1. Preparation of PBS Solution: Standard 0.1 mol of PBS powder was added to 1 liter of double-distilled water, and it was mixed thoroughly to ensure complete dissolution.
2. Preparation of Short Peptide Enteral Nutrition Solution: 4 grams of short peptide enteral nutrition powder was added to 100 milliliters of lukewarm water to prepare a suspension, and it was mixed thoroughly to ensure complete dissolution.

### 2.4 Animals

C57BL/6 mice (male, 8–12 weeks old) from the Laboratory Animal Center of Chongqing Medical University (CQMU, Chongqing, China) were deployed. Before experiments, the mice were adapted to the new environment for a week at 22°C with free access to food and water and with a 12 h light/dark cycle. The Ethics Committee of Dazu District People’s Hospital in Chongqing authorized the study protocol. (Approval No.: 2021LLSC047).

### 2.5 Animal models

Mice were assigned at random into four groups equally (*n* = 3): control (Con), CLP + SPEN1, CLP + SPEN3, and CLP + SPEN7 groups. The mice were placed in a supine position and anesthetized with intraperitoneal 10% chloral hydrate (0.05 mL/10 g). The anesthesia intensity was monitored by pinching the toes with tweezers till they did not respond to needle stimulation. All surgeries were performed under sterile conditions. Mice were routinely prepared and Shaved the lower half of the abdomen with the razor blades, and disinfect the shaved skin area with 70% ethanol. We used a surgical knife to make a longitudinal incision (about 1.5 cm) in the middle of the skin, without directly penetrating the peritoneal cavity on the skin. Once the skin was incised, identified the white fascia in the middle of the abdominal muscle tissue and dissected it. Then, performed an intermuscular incision and incisions on the fascia and peritoneal layers. Used blunt dissection forceps to locate the cecum, ensuring it is exposed. Be careful during the operation, and be sure not to damage the lower small bowel when cutting through the abdominal muscles. Used forceps to locate and forceps the cecum. In most cases, the cecum was located on the left side of the animal (or on the right side when the mouse was on its back), and its position can vary. The middle part of the cecum was ligated with a 3–0 silk suture, and punctured with an 18-gauge needle between the ligations at the distal end of the cecum. Subsequently, a small amount of feces was gently squeezed out of the cecum; afterward, the cecum was sent back to the abdominal cavity to restore its original position; afterward, the abdominal incision was closed. and the mice were returned to their cages. Immediately after surgery, fluid resuscitation was performed by subcutaneous injection of saline (50 mL/kg) at a temperature of 37°C. The mice were euthanized via carbon dioxide box anesthesia on days one, three, and seven after CLP surgery, and ileum tissues and contents were collected and analyzed. From 1 to 7 days after surgery, all CLP + SPEN mice were allowed to eat ad libitum and drink water with 4 g of short peptide. Short peptides are compound preparations, mainly composed of hydrolyzed whey protein, maltodextrin, vegetable oil, minerals, vitamins, and trace elements.

### 2.6 Histopathology

After anesthesia, all mice were sacrificed, and their ileum was dissected out. Fresh ileum tissue was fixed with 4% polyformaldehyde for 48 h, dehydrated in a graded ethanol series, and embedded in paraffin. Standard sections (4–5 μm thick) were cut under a microscope to get optimal orientation and stained with Hematoxylin and Eosin staining (H&E). The stained slides were examined and photographed via an inverted microscope.

### 2.7 H&E staining

1. The ileum tissue was removed from the EP tube, and the 4% paraformaldehyde on the surface of the tissue was repeatedly washed away with PBS.
2. Dehydration: The ileum tissue was immersed in 50% ethanol solution for approximately 5 min, followed by 70% ethanol solution for 5 min, 80% ethanol solution for 5 min, 90% ethanol solution for 5 min, and finally in 100% ethanol solution for 5 min.
3. Clearing: The ileum tissue was sequentially immersed in xylene (I) and (II) for 10 min each to replace the ethanol in the tissue.
4. Embedding: The transparent ileum tissue was fully immersed in liquid paraffin and then placed in a container filled with liquid paraffin. After cooling and solidification, it was removed.
5. Sectioning and Mounting: Using a microtome, paraffin sections with a thickness of 4 micrometers were cut, placed in hot water to flatten, slowly transferred to glass slides, and then baked in a 60°C incubator for 1 h.
6. Dewaxing and Staining: The paraffin sections were sequentially immersed in xylene (II) and (I) for dewaxing, each for 15 min. Subsequently, the sections were placed in a gradient of 100% ethanol for 5 min, 90% ethanol for 5 min, 80% ethanol for 5 min, 70% ethanol for 5 min, and 50% ethanol for hydration, followed by rinsing with running water for 5 min.
7. Staining: The sections were stained in hematoxylin aqueous solution for 1 min. They were then placed in hydrochloric acid-alcohol for differentiation and rinsed with distilled water for 1 h. Finally, eosin staining was performed for 1 min.
8. Dehydration, Clearing, and Mounting: Gradient dehydration was performed using 50, 70, 80, 90, and 100% ethanol, each for 5 min. The sections were then cleared in xylene and mounted using neutral balsam after processing.
9. Observation and Scoring: The tissue sections were observed under a microscope. Images at 100× and 200× magnifications were captured using an upright microscope. Five random fields were selected and photographed for each section. Three individuals performed double-blind scoring of intestinal injury based on Chiu’s scoring system. The scoring criteria included the following six items: (1) Normal intestinal villi (score 1, grade 0);(2) Submucosal space at the villus tip with capillary congestion (score 2, grade 1); (3) Enlarged submucosal space with separation of intestinal mucosa from the submucosa (score 3, grade 2); (4) Separation of mucosa and submucosa extending to both sides of the intestinal villi (score 4, grade 3); (5) Blunted villi with exposure of lamina propria and its blood vessels, and inflammatory tissue infiltration (score 5, grade 4); (6) Digestion and disintegration of lamina propria, hemorrhage, or ulcer formation (score 6, grade 5).

### 2.8 Metabolomics analysis

This part of the experiment was completed at the Mass Spectrometry Center of the International Joint Maternal-Fetal Laboratory, Chongqing Medical University. The specific steps are as follows.

#### 2.8.1 Specimen processing

1. Retrieve 40 mg of ileum tissue and record the weight.
2. Place it in a 2 mL centrifuge tube and add 200 μL of NaOH.
3. Add 200 μL of methanol, vortex for 30s, and then add 20 μL of internal standard.
4. Add three iron beads and use a physical impact disruptor at 30F for 1 min. Note to balance and lock it to prevent imbalance.
5. Then centrifuge at 1200 rpm at 4°C for 15 min and collect 320 μL of supernatant.
6. Transfer to a new tube and add 34 μL of pyridine.
7. Add 20 μL of MCF, vortex for 30s.
8. Add approximately 400 μL of chloroform and vortex for 30s.
9. Add 400 μL of sodium bicarbonate, vortex for 10s, centrifuge at 2,000 rpm for 10 min, and discard the upper and middle layers, retaining the lower layer.
10. Add a small amount of Na2CO3 powder.
11. Aspirate the lower chloroform layer for loading, approximately 200 μL, and label it accordingly, such as ileum C, feces F.

#### 2.8.2 Chromatography (GC)/mass spectrometry (MS) analysis

The derivatized samples were analyzed via a model 7,890 gas chromatograph (Agilent, Santa Clara, CA, United States) with a ZB-1701 capillary column (20 m × 180 μm id × 0.15 μm with a 5-m guard column, Phenomenex, Torrance, CA, United States) connected to an MSD 5975 single quadrupole mass spectrometer (Agilent) in electron ionization mode at 70 eV. The GC and MS procedures adhered to the prescribed protocol of Smart et al. The isolated chloroform phase was injected at 290°C in pulsed splitless mode, using helium carrier gas at a flow rate of 1 mL/min. Additionally, the program temperature was conducted as follows: 45°C for initial temperature, 9°C/min to 180°C for ramped, then 40°C/min to 240°C, and finally 80°C/min to 280°C. Furthermore, the auxiliary temperature was set at 290°C, the quadrupole mass analyzer temperature at 230°C, and the source temperature at 150°C. The mass range was 30–550 m/z, the scan speed was 1.562 m/z units/s, and the solvent delay was 5.5 min.

### 2.9 16S rRNA sequencing

After excretion, mice ileal contents samples were gathered and directly frozen at −80°C. Post-extraction of total ileal DNA contents, the V3-V4 region of the 16S rRNA gene was amplified via primers 338F (5´-ACTCCTACGGGGAGGCAGCA-3′) and 806R (5´-GGACTACHVGGGTWTCTAAT-3′) according to the manufacturer’s instructions. A sequencing connector was added to the primers end, then PCR amplification was conducted and the products were purified, quantified, and homogenized to form sequencing libraries. The constructed libraries were first exposed to library quality control, and the libraries that met the required standards were sequenced by Illumina Novaseq 6,000.

### 2.10 Chromatography (GC)/mass spectrometry (MS) analysis

The derivatized samples were analyzed via a model 7,890 gas chromatograph (Agilent, Santa Clara, CA, United States) with a ZB-1701 capillary column (20 m × 180 μm id × 0.15 μm with a 5-m guard column; Phenomenex, Torrance, CA, United States) connected to an MSD 5975 single quadrupole mass spectrometer (Agilent) in electron ionization mode at 70 eV. The GC and MS procedures adhered to the prescribed protocol of Smart et al. The isolated chloroform phase was injected at 290°C in pulsed splitless mode, using helium carrier gas at a flow rate of 1 mL/min. Additionally, the program temperature was conducted as follows: 45°C for initial temperature, 9°C/min to 180°C for ramped, then 40°C/min to 240°C, and finally 80°C/min to 280°C. Furthermore, the auxiliary temperature was set at 290°C, the quadrupole mass analyzer temperature at 230°C, and the source temperature at 150°C. The mass range was 30–550 m/z, the scan speed was 1.562 m/z units/s, and the solvent delay was 5.5 min.

### 2.11 Statistical analysis

MetaboAnalyst 5.0^1^ was employed for metabolomics studies, and the raw data were summed and normalized. Afterward, log10 transformation and autoscaling were conducted to make the functionality more comparable. Unsupervised PCA was first performed to identify the distribution and separation among groups. A supervised partial least squares discriminant analysis (PLS-DA) was used to maximize the classification difference. In addition, R2 and Q2 were deployed to determine the prediction accuracy and predictive power of the PLS-DA model. Based on the PLS-DA model, differential metabolites were recognized by combining the FDR using the *t*-test and the variable importance projection variable important for the Projection (VIP) values to identify differential metabolites. The queue values were set as VIP > 1, *p* < 0.05, and FC > 1.2 or < 0.8. GraphPadPrism8 (GraphPadSoftware, USA) and SPSS22.0 (IBM, USA) one-way analysis of variance (ANOVA) were deployed to evaluate comparisons between several groups, and Tukey’s *post-hoc* test was utilized as a post-hoc test to compare the variances between the two groups. In addition, Pearson’s r coefficient was conducted to calculate Bivariate correlations. The R software (v4.1.2) was employed to construct the heat maps, and Spearman’s correlation analysis was deployed to explore the link between GM and serum metabolites. *p* < 0.05 was set as statistically significant.

## 3 Results

### 3.1 The impact of short peptide nutritional preparations on intestinal barrier function in mice

**Figure 1:**
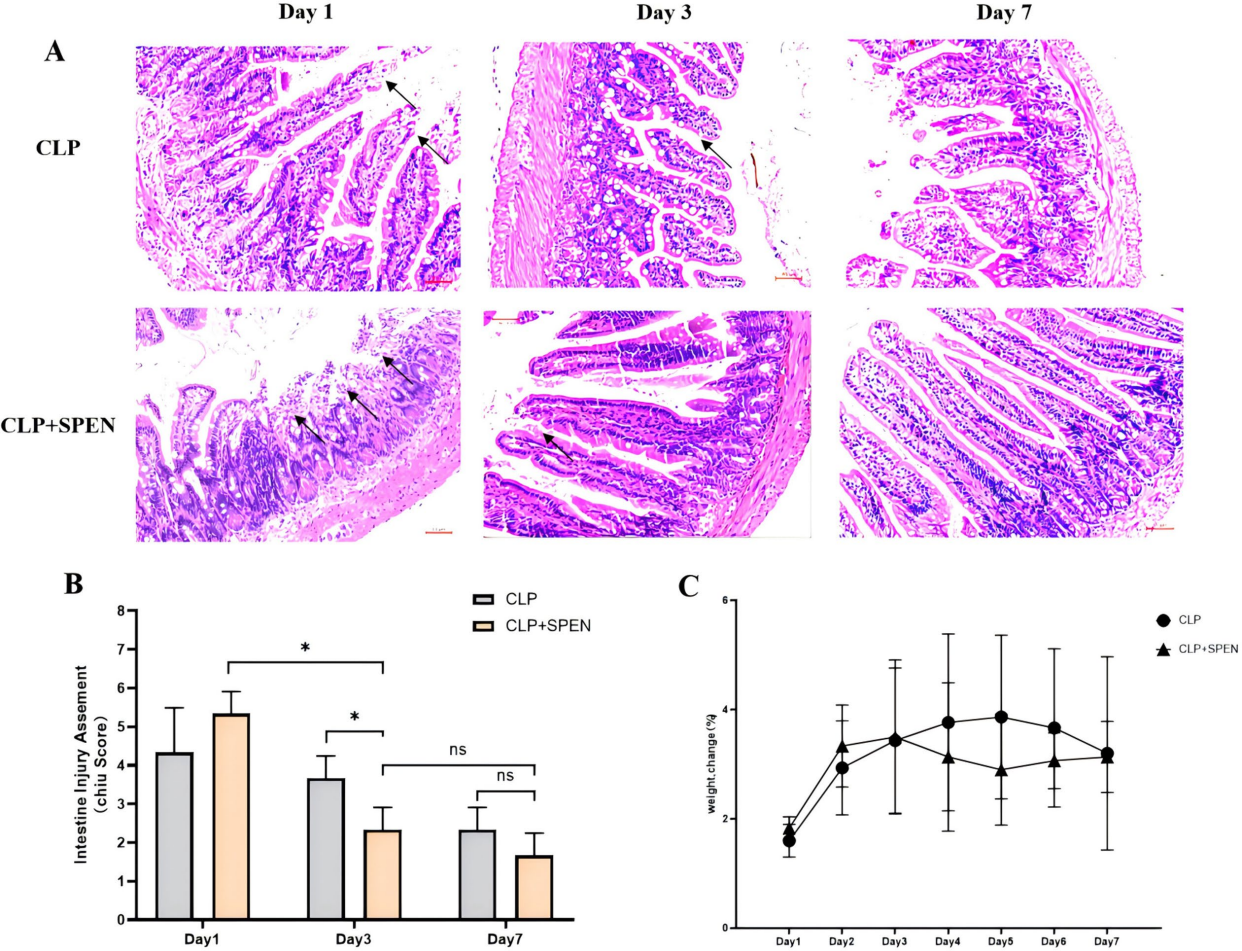
**(A)** H&E staining images of intestinal mucosa in CLP and CLP + SPEN groups. **(B)** Quantification of intestinal injury by Chiu’s score. (*p < 0.05; ns, no significant). **(C)** Trend plot of body weight change in CLP and CLP + SPEN groups over 7 days.

CLP surgery was deployed to assess the therapeutic impacts of short peptides onseptic mice. The mice intestinal mucosa in the CLP + SPEN groups was significantly diverse from that in the CLP group on the first, third, and seventh days. The images displayed that CLP surgery resulted in erosion of upper villous surfaces and extensively destructed intestinal villi. Short peptide treatment maintained the intestinal structure and attenuated the histopathological changes in septic mice (Figure 1A). Consistent with the intestine histological changes, Chiu’s score in the short peptides-treated group was reduced compared with that in the CLP group (Figure 1B). The CLP and the CLP + SPEN groups lost weight in the first 3 days but with no significant difference, and after the fourth day, the CLP + SPEN group showed an advantage in weight recovery (Figure 1C).

### 3.2 The change in intestinal microbiome abundance

**Figure 2:**
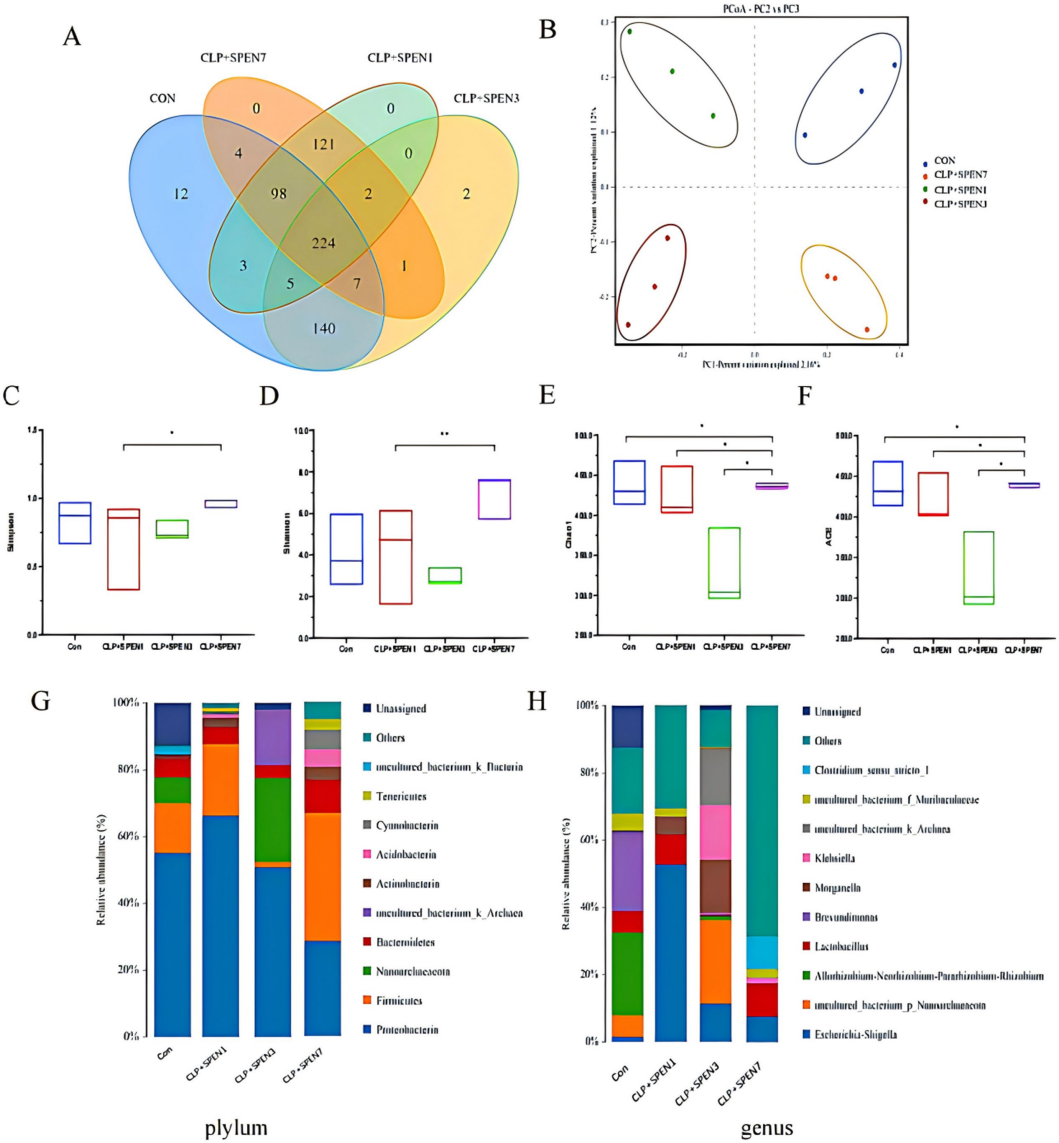
Intestinal microbiome abundance changes. **(A)** OTU Venn of the difference in the OTU numbers in all groups. **(B)** OPLS-DA-based analysis of differences among all groups. **(C–F)** The difference in diversity indexes between groups **(G,H)** Intestinal microbial composition at the phylum and genus levels, respectively, in all groups.

The Wayne plots (Figure 2A) present 224 Operational Taxonomic Units (OTUs) overlapped in all groups, 231 OTUs overlapped in the CLP + SPEN1 and CLP + SPEN3 groups, and two different bacteria were observed in CLP + SPEN3, while no different bacteria were recognized in CLP + SPEN1 and CLP + SPEN7.To further clarify the impact of short peptide treatment on the diversity of mice,a and *β* diversity analyses were performed in this study, and the β diversity of intestinal microorganisms was evaluated via PCoA (Figure 2B). Significant variances were recognized between the four groups of well-isolated bacteria in terms of the Shannon, chao index, ACE index, and Simpson index (Figures 2C–F). Bacterial distribution and abundance were then measured at individual levels (Figures 2G–F). Ten bacterial species were monitored at the Phylum level, enriched for Proteobacteria, Firmicutes, Nanoarchaeaeota, Bacteroidetes, Actinobacteria, uncultured_bacterium_k_Archaea, Acidobacteria, Cyanobacteria, Tenericutes, uncultured_bacterium_k_Bacteria. Among them, Proteobacteria, Firmicutes, Bacteroidetes, Actinobacteria, Cyanobacteria Tenericutes(Proteobacteria, Firmicutes, Bacteroidetes, Actinobacteria, Acidobacteria, Cyanobacteria, Tenericutes). It has statistical significance (Figure 2G), at the Genius level, It enriched Escherichia-Shigella, uncultured_bacterium_p_Nanoarchaeaeota, Allorhizobium-Neorhizobium-Pararhizobium-Rhizobium, Lactobacillus, Brevundimonas, Morganella, Klebsiella, uncultured_bacterium_k_Archaea, uncultured_bacterium_f_Muribaculaceae, Clostridium _sensu_stricto_1, Among them, Lactobacillus and uncultured_bacterium_f_Muribaculaceae showed specific changes (Figure 2H).

### 3.3 Short peptides effect on gut flora abundance

**Figure 3:**
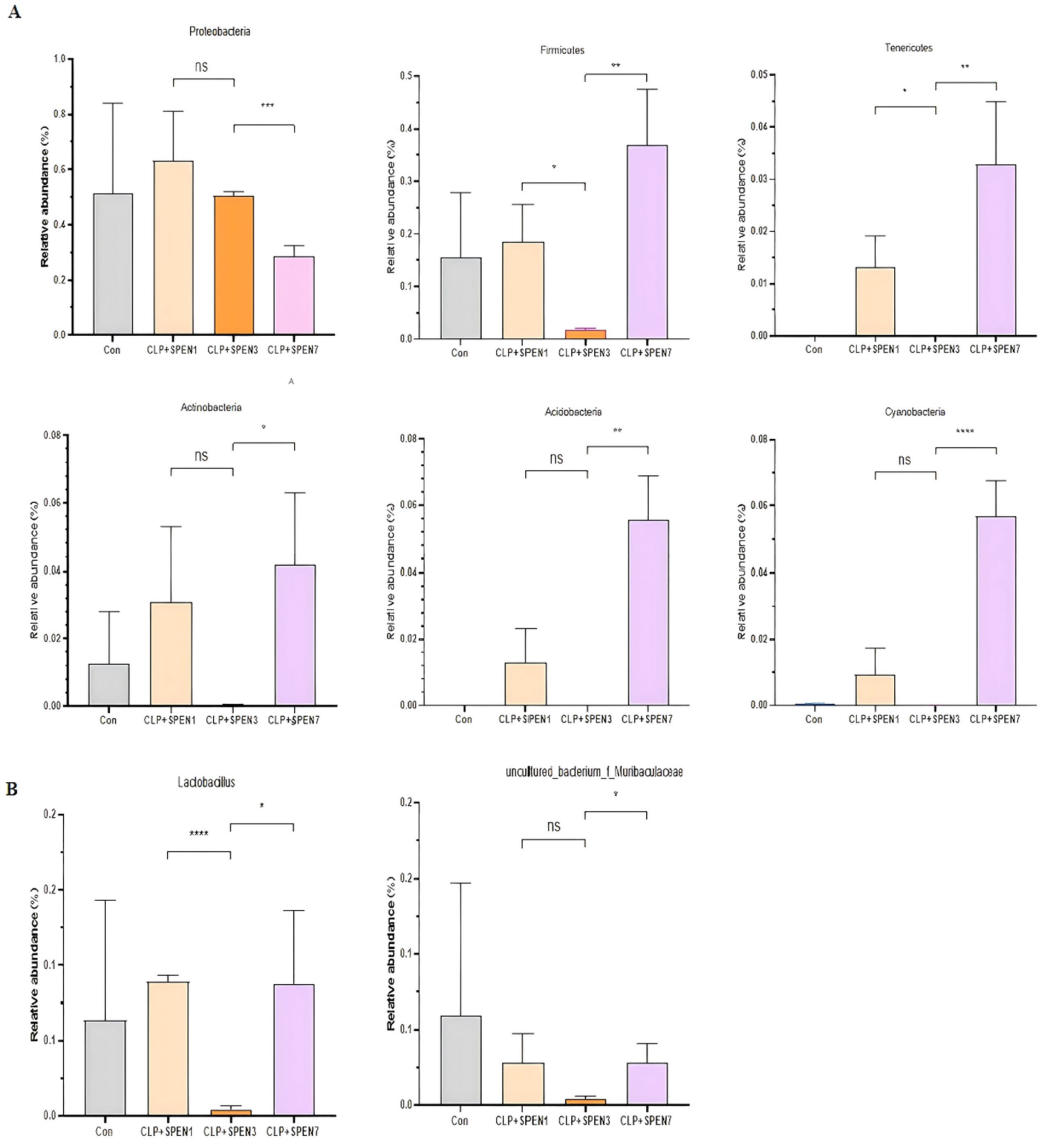
(**A,B**) The relative abundance changes of meaningful intestinal flora (genus and phylum levels, respectively) in four groups(*p < 0.05, **p < 0.01, ***p < 0.001, ****p < 0.0001; ns, no significant).

The Proteobacteria relative abundance was gradually decreased, with the lowest abundance recognized in the CLP + SPEN7 group. The abundance of Firmicutes, Bacteroidetes, Actinobacteria, Acidobacteria, Cyanobacterium, and Tenericutes was significantly lowered in the CLP + SPEN3 group and then gradually raised. The abundance increased in the CLP + SPEN7 group, and all revealed statistical significance (Figure 3A). The Lactobacillus abundance was significantly lowered in the CLP + SPEN3 group and gradually elevated after the third day. The abundance increased in both CLP + SPEN7 groups. The uncultured_ubacterium_f_Muribaculaceae abundance elucidated no statistically significant variation between the CLP + SPEN1 and the CLP + SPEN7 groups. Comparing the CLP + SPEN3 and the CLP + SPEN7 groups, the results elucidated that the unculturedubacterium_f-Muribaculaceae abundance was gradually raised between the third and seventh days after short peptide treatment, with statistical significance (Figure 3B).

### 3.4 Analysis of mice intestinal metabolome

#### 3.4.1 Analysis of metabolites in mice intestinal tissue

**Figure 4:**
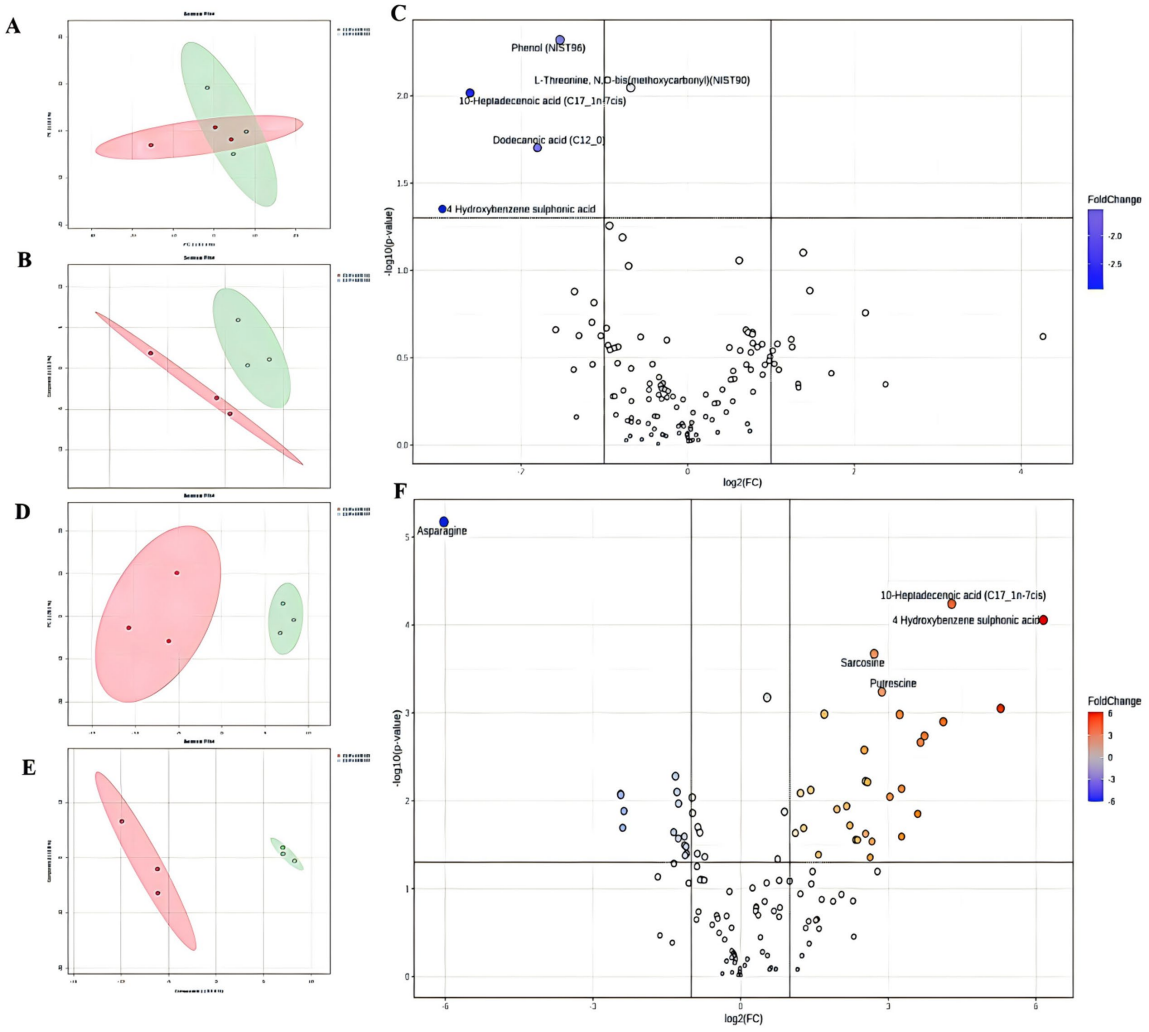
**(A, B)** Scores plots of PCA and OPLS-DA between CLP+SPEN1 and CLP+SPEN3 groups. **(C)** Volcano Plots coefficient between CLP+SPEN1 and CLP+SPEN3 groups. **(D, E)** Scores plots of PCA and OPLS−DA between the CLP + SPEN3 and CLP +SPEN7 groups. **(F)** Volcano Plots coefficient between CLP+SPEN3 and CLP+SPEN7 groups.

In total, 68 critical metabolites were found in the CLP + SPEN3 and CLP + SPEN7 groups; 47 showed elevation, and the remaining 21 demonstrated a decrease. Typically, we recognized 45 diverse metabolites with statistical significance (Figure 4). More specifically, 30 showed an increase, and 15 revealed a decrease (Table 1). When comparing the CLP + SPEN1 and CLP + SPEN3 groups, we identified four diverse metabolites with statistical significance. Furthermore, only four reduced metabolites were observed (Table 1).

**Table 1:** The statistical table on the number of differential metabolites.

Group name	Total sig metabolites	Upregulated	Downregulated
SPEN1 vs. SPEN3	4	0	4
SPEN3 vs. SPEN7	45	30	15

#### 3.4.2 Discovery of the role of 10-heptadecanoic and dodecanoic acids in intestinal metabolism

**Figure 5:**
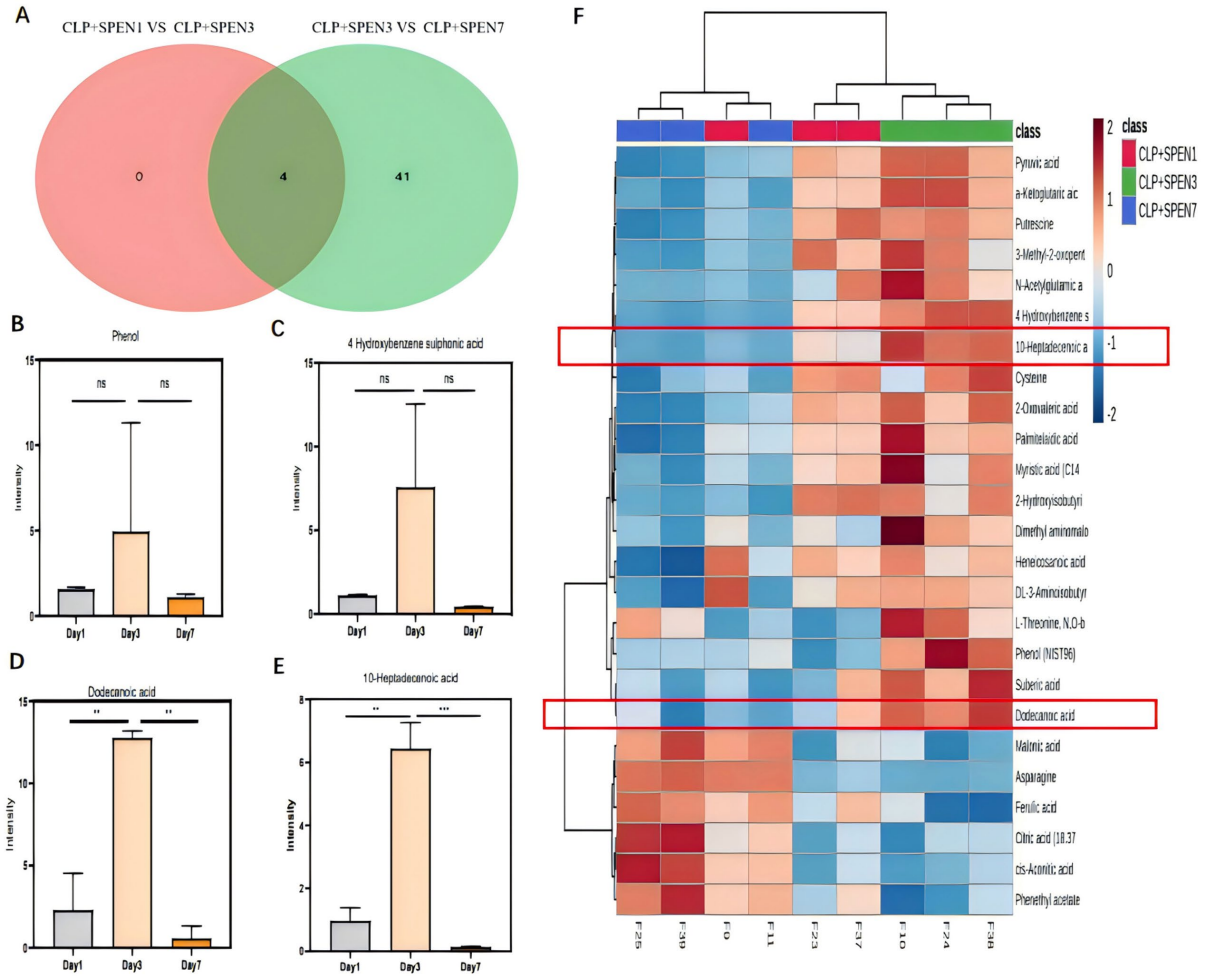
**(A)** Venn diagram of differential metabolites. CLP + SPEN1 vs. CLP + SPEN3 (red) and CLP + SPEN3 vs. CLP + SPEN7 (blue, *n* = 3/group). **(B–E)** Histogram of metabolites distribution. **(F)** Heat map of differential metabolites between CLP + SPEN1, CLP + SPEN3, and CLP + SPEN7 groups. (Red refers to high levels, blue refers to low levels).

Figure 5A shows a differential Venn diagram of four metabolites that were commonly different.10-heptadecanoic and dodecanoic acids had statistical significance (Figures 5B–E). The heat maps displayed the trend of change, where the two metabolites were raised in the CLP + SPEN3 group, while they were decreased over time in the CLP + SPEN7 group after one week (Figure 5F).

### 3.5 Short peptides can change the composition of the intestinal microecology and regulate certain metabolites in the body

**Figure 6:**
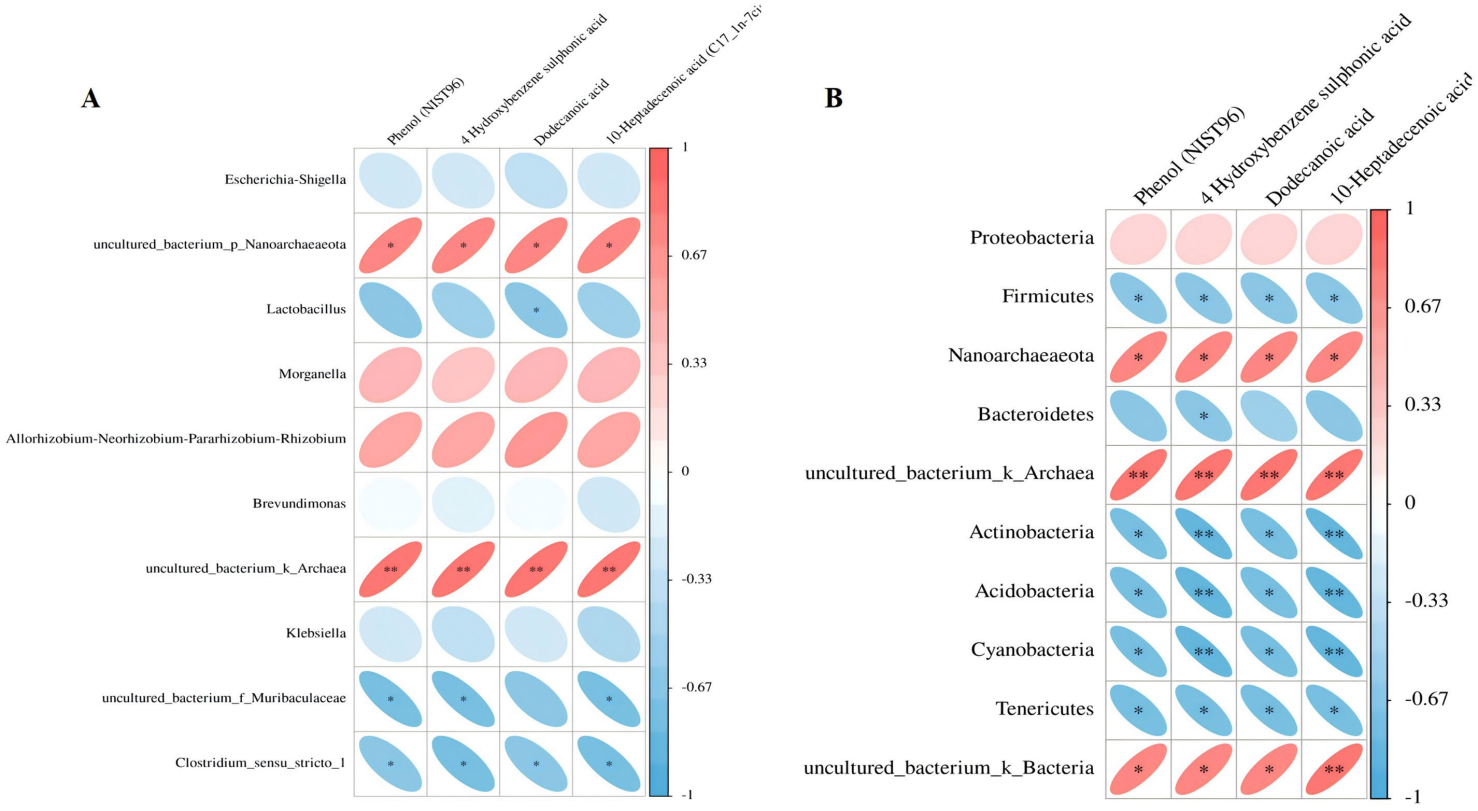
**(A,B)** The correlation heat map analysis between specific metabolites and microbial microbiome (below refers to the variant metabolite; the right represents the various species; the color depth refers to the association size, the negative correlation in blue, the positive correlation in red, *p < 0.05, **p < 0.01).

Spearman correlation was conducted to explore the relation between differential serum metabolites and microbiome at the genus and phylum levels in the treated mice. At the generic level, Escherichia-Shigell, uncultured_bacterium_p_Nanoarchaeaeota, Allorhizobium-Neorhizobium-Pararhizobium-Rhizobium, Brevundimonas, Morganella, Klebsiella, uncultured_bacterium_k_Archaea, uncultured_bacterium_f_Muribaculaceae, and Clostridium_sensu_stricto_1 were linked to most of the metabolites (Figure 6A). The dodecanoic acid was negatively correlated to Lactobacillus; the 10-heptadecenoic acid was negatively correlated to uncultured_bacterium_f_Muribaculaceae. At the phylum level, Proteobacteria, Firmicutes, Nanoarchaeaeota, Bacteroidetes, Actinobacteria uncultured_bacterium_k_Archaea, Acidobacteria, Cyanobacteria, Tenericutes uncultured_bacterium_k_Bacteria were correlated to most of the metabolites (Figure 6B). The 10-heptadecenoic and dodecanoic acids were negatively correlated with Firmicutes, Actinobacteria, Acidobacteria, Cyanobacteria, and Tenericutes. The results indicate that short peptides can change the intestinal microecology composition and regulate certain metabolites in the body.

## 4 Discussion

Sepsis is a complex disease identified by a life-threatening organ dysfunction in critically ill patients. It may be caused by a primary infectious cause or by a secondary infection of damaged tissues. This condition poses a major challenge in the management of critically ill patients and requires timely and effective intervention to prevent further deterioration of the condition (13). Despite significant developments in diagnostic and therapeutic procedures, mortality rates for sepsis patients remained unchanged from the 1960s to the late 1990s. Sepsis constitutes unique challenges due to its multilevel pathogenesis involving molecular, cellular, and organ systems. Ecological dysregulation in sepsis patients is characterized by reduced microbial variety and overgrowth of opportunistic pathogens (e.g., Enterobacteriaceae, Enterococci, and Staphylococci), and this ecological dysregulation can lead to elevation of intestinal permeability and systemic infections, impacting distant organ function and systemic immunity (14). Among other things, the homeostasis of the GM interacting with the intestinal epithelial barrier, which is necessary for preserving normal gut function, is significantly disrupted in sepsis.

Early enteral nutritional support protects the integrity of the gastrointestinal mucosa and enhances the immune function of patients (15). It also stimulates the release of immunoglobulin A (IgA) from gut-associated lymphoid tissue (GALT), which reduces bacterial adhesion to epithelial cells and prevents increased intestinal permeability (16). Short peptide enteral nutrition is formulated as a pre-digested formula that improves the tolerance of enteral nutrition and improves nutritional levels. According to current guidelines for the treatment of sepsis, early recognition and appropriate management of sepsis in the first few hours after its onset can improve prognosis (17). Dysbiosis of intestinal flora may increase host susceptibility to disease; however, the intestinal flora and metabolite mechanisms in sepsis are unclear. There are few studies to improve sepsis from the therapeutic point of view of intestinal flora and metabolites; accordingly, we used a mouse model of sepsis and found that the emergence of intestinal flora and metabolites in a significant difference during short peptide treatment can improve the intestinal damage in mice, which can provide a certain new therapeutic strategy in the process of future clinical practice.

Sepsis severely affects the GM composition and metabolic changes, which in turn may lead to organ dysfunction (18). A common cause of sepsis is intestinal perforation. Our experiment replicated the experimental method of Mishra SK et al. for sepsis modeling (19). The images displayed that CLP surgery resulted in erosion of upper villous surfaces and extensively destructed intestinal villi. Short peptide treatment maintained the intestinal structure and attenuated the histopathological changes in septic mice. Consistent with the intestine histological changes, Chiu’s score in the short peptides-treated group was reduced compared with that in the CLP group.

According to our findings, we can conclude that short peptide nutritional preparations can significantly enhance the intestinal mucosal barrier function and attenuate inflammatory cell infiltration. According to many studies, intestinal microorganisms have a central function in sepsis development. Lactobacilli, especially Lactobacillus spp., are also present in the intestinal tract and have anti-inflammatory properties, preventing episodes of colitis and reversing established experimental intestinal inflammation (20). *In vitro* and *in vivo* studies have revealed that prolonged lactobacilli administration stimulates qualitative and quantitative alterations in the human gastrointestinal microbial ecosystem with encouraging perspectives in counteracting pathology-associated physiological and immunological changes (21).

Our study conducted high-throughput sequencing through 16sRNA analysis technology to observe the diversity and structure of the intestinal microbiota in mice with sepsis and after short peptide intervention. The results found that both the abundance and diversity of intestinal microbiota in septic mice were significantly reduced. In the *α*-diversity analysis, the Simpson, Shannon, Chao, and ACE indices in the CLP + SPEN7 group were the highest among all groups, indicating the best abundance and maximum diversity of intestinal microbiota after 7 days of short peptide intervention. Although the intestinal microbial community diversity in the CLP + SPEN7 group was higher than that in the Con group, there was no significant difference between the two groups, suggesting that the short peptide nutritional formulation may have slightly increased diversity over time. No statistical significance was found between the two groups, which could be due to the inability of intestinal microbiota diversity to return to normal levels after treatment. The richness and diversity of intestinal microbiota in mice with sepsis were greatly affected. The Chao and ACE indices in the CLP + SPEN7 group were higher than those in the Con group, CLP + SPEN1 group, CLP + SPEN3 group, and CLP + SPEN7 (comparing within different time points of SPEN intervention) group, indicating that the addition of short peptide nutritional formulation improved the community richness of intestinal microbiota, which was significantly related to the duration of short peptide administration. The intestinal microbiota richness and diversity were highest on the seventh day after short peptide administration in mice. We speculate that the role of short peptides may be to improve sepsis symptoms by increasing the abundance and diversity of intestinal microbiota and correcting the dysfunctional structure of intestinal microbial communities.

The largest microbial community in humans exists in the gut. The most common were Bacteroidetes, Firmicutes, and Actinobacteria (22). At the phylum level, Bacteroidetes and Firmicutes were also the two most abundant microbial communities in the gut of mice. The gut microbiota has a positive impact on lipid metabolism by inhibiting lipoprotein lipase activity in adipocytes. In addition, Bacteroides can improve the efficiency of lipid hydrolysis by upregulating the expression of pancreatic lipase in *Escherichia coli*, which is required for lipid digestion (23). Magne’s team’s research indicates that the relationship between the two dominant phyla, expressed as the Firmicutes/Bacteroidetes ratio, has been associated with several pathological conditions (24). Our study found that there were changes in the microbiota of *Pseudomonas aeruginosa* and other bacteria such as Firmicutes in septic mice. However, after short peptide intervention, the relative abundance of *Pseudomonas aeruginosa* gradually decreased, and the lowest abundance was observed on the 7th day after intervention; The phyla Firmicutes, Bacteroidetes, Actinobacteria, Acinetobacteria, Cyanobacteria, and Actinobacteria gradually increased, but their abundance decreased to its lowest on the third day and then gradually increased, reaching its highest on the seventh day. Experiments have shown that short peptide intervention may restore the structure of the gut microbiota by increasing the abundance of Firmicutes, Firmicutes, Bacteroidetes, Actinobacteria, Acinetobacteria, Cyanobacteria, and Actinobacteria, thereby inhibiting the pathogenic role of Proteobacteria.

At the genus level, there was an enrichment of Escherichia-Shigella, uncultured_bacterium_p_Nanoarchaeaeota, Allorhizobium-Neorhizobium-Pararhizobium-Rhizobium, Lactobacillus, Brevundimonas, Morganella, Klebsiella, uncultured_bacterium_k_Archaea, uncultured_bacterium_f_Muribaculaceae, and Clostridium_sensu_stricto_1. Among them, Lactobacillus and uncultured_bacterium_f_Muribaculaceae exhibited specific changes. The abundances of Lactobacillus and uncultured_bacterium_f_Muribaculaceae reached their lowest points on the third day after short peptide treatment intervention, and then gradually recovered, restoring their abundances by the seventh day. Lactobacillus, a well-known genus of beneficial bacteria in the intestinal tract, has a protective impact on the intestinal tract in colitis (25). Lactobacilli colonize the intestinal tract and can regulate the intestinal barrier function by balancing the intestinal flora in the host body (26). It has been found that Lactobacillus can activate NF-kB and STAT signaling pathways and enhance the production and release of interferon-gamma (IFN-*γ*) and interleukin 12 (IL-12) from antigen-presenting cells, which can synergistically stimulate the cytotoxic T lymphocytes (CTL) proliferation and differentiation and enhance the cellular immune response development. Therefore, Lactobacillus has an anti-inflammatory function by regulating host intrinsic immunity (27). We can conclude that the upregulation of Lactobacillus abundance may be a potential biomarker for short peptide treatment of sepsis, providing new insights for subsequent research.

Gut flora metabolites regulate immunity, preserve the intestinal environment, and supply the body with energy (28), such as threonine (29) and tryptophan (30) can regulate the tight junction protein expression and improve LPS-induced intestinal barrier dysfunction. In our study, metabolites such as 10-heptadecenoic and dodecanoic acids, among others, were elevated and then decreased with the day seven change in short peptide treatment.

Microbial-derived metabolites regulate immune homeostasis. Dodecanoic acid (DA), also known as LA (Lauric acid), is abundant in coconut oil along with palmitic acid (PA). DA has been shown to possess anti-microbial properties against various microbial pathogens against a broad range of bacteria, fungi and viruses (31). It was a fatty acid with 12 carbon atoms, is absorbed into intestinal epithelial cells via passive diffusion and directly initiates *β*-oxidation in the mitochondria in a carnitine-independent manner (32). It is also associated with agonists of Toll-like receptors, with Toll-like receptor 4 signaling increasing the risk of colitis and colitis-associated cancer. These metabolites may play a role in improving symptoms and inflammation (33). Their results showed a strong *in vitro* antagonism of Oxacillin with LA or LA-rich palm oil, suggesting that administering these drugs concurrently, at higher, may negatively impact their pharmacological properties in the treatment of *Staphylococcus aureus* (34).

In this study, a joint analysis of gut microbiota and differential metabolites was conducted. At the genus level, the metabolite Dodecanoic acid decreased with an increase in the abundance of Lactobacillus, while 10-Heptadecenoic acid decreased with an increase in the abundance of uncultured_bacterium_f_Muribaculaceae. At the phylum level, both 10-Heptadecenoic acid and Dodecanoic acid decreased with an increase in the abundance of Firmicutes, Actinobacteria, Acidobacteria, Cyanobacteria, and Tenericutes. Among them, Dodecanoic acid and 10-Heptadecenoic acid were two metabolites with differential significance. Correlation analysis results showed that during the short-peptide treatment process, the abundance of Lactobacillus and uncultured_bacterium_f_Muribaculaceae gradually increased on the 7th day, accompanied by a significant decrease in the metabolites Dodecanoic acid and 10-Heptadecenoic acid, which improved intestinal injury and alleviated sepsis in mice. In summary, Lactobacillus can be suggested as a probiotic for the treatment of sepsis.

## 5 Conclusion

In summary, this experiment investigated the relationship between sepsis and the gut microbiota before and after short peptide intervention. Due to the relatively small number of experimental animals used in this study and the diverse nature of gut microbiota, we were unable to fully reconstruct the intestinal ecological environment of sepsis patients. However, through the analysis and comparison of the gut microbiota of mice in the CLP + SPEN group and the Con group, we found that sepsis mice exhibited an imbalance in their gut microbiota. The study revealed that 7 days of short peptide intervention increased the growth of beneficial bacteria Lactobacillus and uncultured_bacterium_f_Muribaculaceae, reduced intestinal metabolites such as Dodecanoic acid and 10-Heptadecenoic acid, enhanced the diversity and abundance of gut microorganisms, altered the structure of the gut microbiota, and maintained the dynamic balance of the flora, thereby playing an important therapeutic role in the treatment of sepsis. We can speculate that Lactobacillus can serve as a probiotic for the treatment of sepsis. Nevertheless, due to the limited number of experimental samples, the exact mechanism of action of short peptides awaits further confirmation by targeted metabolomics.

## Data Availability

The original contributions presented in the study are included in the article/supplementary material, further inquiries can be directed to the corresponding authors.

## Footnotes

^1^ https://www.MetaboAnalyst.ca
